# The CpG Landscape of Protein Coding DNA in Vertebrates

**DOI:** 10.1111/eva.70101

**Published:** 2025-05-04

**Authors:** Justin J. S. Wilcox, James Ord, Dennis Kappei, Toni I. Gossmann

**Affiliations:** ^1^ Computational Systems Biology, Faculty of Biochemical and Chemical Engineering TU Dortmund University Dortmund Germany; ^2^ Organismal and Evolutionary Biology Research Program, Faculty of Biological and Environmental Sciences University of Helsinki Helsinki Finland; ^3^ Cancer Science Institute of Singapore National University of Singapore Singapore Singapore; ^4^ NUS Center for Cancer Research, Yong Loo Lin School of Medicine National University of Singapore Singapore Singapore; ^5^ Department of Biochemistry, Yong Loo Lin School of Medicine National University of Singapore Singapore Singapore

**Keywords:** base composition, dinucleotides, DNA methylation, epigenetics, protein coding DNA

## Abstract

DNA methylation has fundamental implications for vertebrate genome evolution by influencing the mutational landscape, particularly at CpG dinucleotides. Methylation‐induced mutations drive a genome‐wide depletion of CpG sites, creating a dinucleotide composition bias across the genome. Examination of the standard genetic code reveals CpG to be the only facultative dinucleotide; it is however unclear what specific implications CpG bias has on protein coding DNA. Here, we use theoretical considerations of the genetic code combined with empirical genome‐wide analyses in six vertebrate species—human, mouse, chicken, great tit, frog, and stickleback—to investigate how CpG content is shaped and maintained in protein‐coding genes. We show that protein‐coding sequences consistently exhibit significantly higher CpG content than noncoding regions and demonstrate that CpG sites are enriched in genes involved in regulatory functions and stress responses, suggesting selective maintenance of CpG content in specific loci. These findings have important implications for evolutionary applications in both natural and managed populations: CpG content could serve as a genetic marker for assessing adaptive potential, while the identification of CpG‐free codons provides a framework for genome optimization in breeding and synthetic biology. Our results underscore the intricate interplay between mutational biases, selection, and epigenetic regulation, offering new insights into how vertebrate genomes evolve under varying ecological and selective pressures.

## 1 Introduction

Mutation rates can vary across vertebrate genomes due to the impacts of epigenetic modifications, their effects on DNA repair mechanisms, and the accessibility of the DNA to mutagens (Cooper and Krawczak 1989; Holliday and Grigg 1993; Bestor 2000; Jjingo et al. 2012). Methylation of cytosine to form 5‐methyl‐cytosine (5mC) represents one of the most common forms of epigenetic modification. This type of modification, however, may lead to spontaneous deamination of cytosine to thymine, resulting in higher mutation rates (Yi 2007; Schübeler 2015). Also, other types of mutational biases with CpG methylation have been reported (Tomkova and Schuster‐Böckler 2018) such as cytosine to guanine mutations in certain types of cancers (Tomkova et al. 2016). The effect of CpG dependent mutations is highly context‐dependent as methylation of cytosine occurs primarily in particular base motifs and genomic regions, and is also likely variable based on other impacts of genomic architecture (Xia et al. 2012; Zhou et al. 2020). As such, the persistence of such methylation targets in the context of high mutation rates may provide insights into the fitness benefits of epigenetically variable sites and associated base motifs.

In vertebrate genomes, DNA methylation primarily occurs on cytosine bases in CpG dinucleotides (a cytosine followed by a guanine in the 5′–3′ direction) and is of significant importance for genome stability (Chen et al. 1998). However, not all CpG dinucleotides in vertebrate genomes are methylated. Instead, DNA methylation is highly context‐dependent, and its distribution can vary across different genomic regions and cell types. The distribution of CpG sites is expected to vary across most genomes (Box 1) based on observations of heterogeneity in base composition (Bernardi et al. 1988; Nabholz et al. 2011) as well as strand biases on the particular complement of the DNA helix observed (CG vs. GC), both of which are well documented in mammals and birds (Evans 2008). In general, CpG dinucleotides within gene promoters, enhancers, and other regulatory elements tend to be infrequently methylated or completely unmethylated, whereas CpG dinucleotides within gene bodies, repetitive elements, and intergenic regions can be more heavily methylated (Laine et al. 2016; Derks et al. 2016). Comparing different vertebrate species while focusing on specific organs highlighted a strong connection between DNA methylation and tissue types (Klughammer et al. 2023). Additionally, analyzing the DNA methylation patterns at gene promoters across species revealed evolutionary differences in methylation patterns for orthologous genes (Klughammer et al. 2023). Together, these findings highlight the importance of CpG sites in regulating epigenetic differences within organisms and across species.

BOX 1 **Expected CpG frequency.**

If there is no context effect, the expected frequency of CpG dinucleotides is dependent on the base composition of DNA (i.e., here denoted as G + C frequency) and the strand bias of C and G nucleotides (Karlin and Mrázek 1997) (Here denoted as G/(G + C), which represents the proportion of G nucleotides out of G + C nucleotides on a single strand). High GC DNA regions often vary in their CpG content based on C and G strand bias. As proteins are encoded on one strand, such bias plays a particularly important role in protein coding DNA.
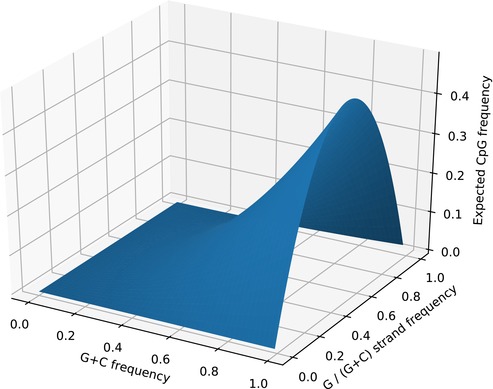

DNA methylation within gene bodies is ubiquitous across vertebrate species, including mammals, birds, reptiles, amphibians, and fishes, although the extent and patterns of this phenomenon may vary across taxa (Zemach et al. 2010; Long et al. 2013). In contrast to the extensively studied promoter methylation, the role of gene body methylation remains poorly understood, partly due to inconsistencies in its patterns across different species. Certain aspects of gene body methylation, such as its association with gene expression and alternative splicing, are also evolutionarily conserved across vertebrates (Anastasiadi et al. 2018). In human cells, gene body methylation levels are often positively correlated with gene expression (Lister et al. 2009; Moore et al. 2012). However, the impacts of gene body methylation on expression are less consistent: methylation is negatively correlated with gene expression levels in avian (Laine et al. 2016; Boman et al. 2024) and murine (Guo et al. 2014) tissues, but positively correlated in human neuron tissue (Lister et al. 2009). These mixed patterns of correlation suggest that gene body methylation may play a role in promoting or suppressing transcription and thereby regulating gene expression levels (Maunakea et al. 2010; Jones 2012). Together these findings highlight the functional significance and potential differential selective pressures acting to maintain gene body methylation.

Gene bodies are of course heterogeneous and (usually) consist of coding and noncoding components. Just as the distribution of CpGs across the genome is nonrandom, the nucleotide composition of coding DNA is nonrandom because of the coding information preserved and because of preferences for certain codons (Fedorov et al. 2002). In yeasts, adjacent codons may have an influence on translation efficiency, suggesting that specific combinations of neighboring codons play a significant role in modulating translation rates, thereby affecting protein expression levels (Gamble et al. 2016). There are biases in codon pair preferences and avoidance across bacteria, archaea, and other eukaryotes (Tats et al. 2008), including CpG‐containing dicodons that are underpresented (nnCGCn and UUCGnn).

The effect of selection on mutations in protein coding DNA is a common measure used to infer the rate of molecular evolution in protein coding genes and the rate of adaptation (Gossmann et al. 2012), for example in birds (Laine et al. 2016; Gossmann et al. 2014). This is because within the genetic code, there are two major types of point mutations, synonymous mutations that do not change the coding amino acid and nonsynonymous mutations which do change the coding amino acid. The ratio of the fixation rate of these two mutation types (dN/dS) holds information on the long‐term selective pressures acting at the amino acid level (Jeffares et al. 2014). However, attempts to infer the rate of molecular evolution and the strength of selection are usually hampered by the action of biased gene conversion (Gossmann et al. 2018; Bolvar et al. 2019). Biased gene conversion is effective at heterozygous sites that contain G/C and A/T, that is, G/A, G/T, C/A, C/T as they tend to be repaired in favor of the G/C base (Duret and Galtier 2009; Kostka et al. 2012). Furthermore, biased gene conversion interacts with features of genomic base composition and mutation rate bias (Subramanian and Kumar 2003). Although a few studies do explore connections between DNA methylation and base composition (for instance, Danchin et al. (Danchin et al. 2011) or the recent finding by Marshall et al. (Marshall et al. 2023) linking codon degeneracy and methylation in bumblebees), comprehensive research focusing on coding regions remains limited. Much of the existing work relies on whole‐genome approaches without specifically dissecting how methylation might shape codon usage or nucleotide composition in protein‐coding sequences.

There are a number of consequences of DNA methylation on the DNA base composition of vertebrate genomes (Holliday and Pugh 1975; Riggs 1975; Pelizzola and Ecker 2011). One of the most severe consequences is a genomic bias in dinucleotide composition that is largely driven by deamination of cytosines in CpG sites. This effect is pronounced in vertebrate genomes, which are deficient in CpG sites compared to the dinucleotide frequency expected from single nucleotide base abundance (Lander et al. 2001). Implications of DNA methylation on genes are usually considered in light of gene expression, and focus on DNA methylation of transcription start sites and gene bodies (Laine et al. 2016; Boman et al. 2024). Insights into CpG dynamics may inform critical evolutionary applications, for example to improve the management of wild populations and enhance breeding programs for food production (Rey et al. 2019; Powell et al. 2023). For instance, in conservation genomics, CpG‐rich regulatory genes may serve as markers for adaptive potential, aiding in the development of strategies to protect species facing environmental pressures. Similarly, in artificial selection, understanding CpG content can help refine genome editing and breeding approaches by optimizing genetic stability and stress response traits. However, the evolutionary implications of DNA methylation on base composition in coding DNA are much less understood.

To address this important knowledge gap, here, we combine the implications of DNA methylation on DNA base composition and its consequences on the rate of molecular evolution of protein coding DNA. For this, we dissect the relationship of the genetic code and CpG methylation and conduct empirical genomic analyses. We examine the necessity and capacity for avoidance of CpG in the standard genetic code. Furthermore, we investigate the occurrence of CpG within protein coding DNA by analysing six vertebrate genomic datasets. Specifically, we addressed the following questions by empirical analyses:

1. Is the CpG content different in coding relative to noncoding genomic regions?
2. Is there variation in CpG content within protein coding DNA of single genes?
3. Are CpG containing dicodons over‐ or underrepresented in protein coding DNA?
4. Is there a functional enrichment of genes with high/low CpG content in their coding DNA?

## 2 Theoretical Motivation

### 2.1 The Genetic Code and CpG Dinucleotides

**FIGURE 1:**
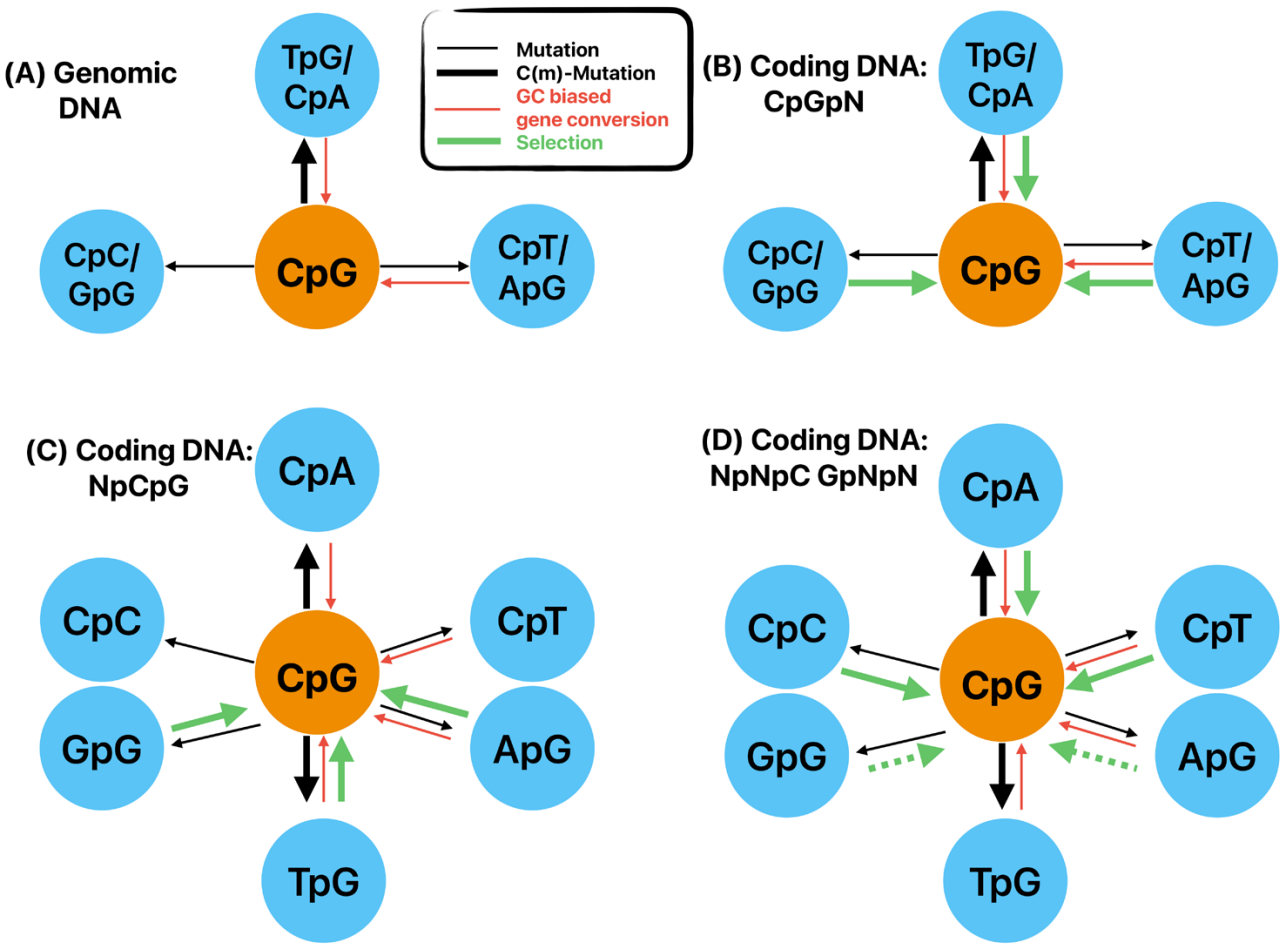
Evolutionary forces acting on CpG dinucleotides. (A) CpG sites in a genomic context (B–D) CpG sites in coding DNA context. C(m)‐Mutation denote a methylation dependent mutation rate. Dashed arrows denote context dependent selection (see Table 2).

**FIGURE 2:**
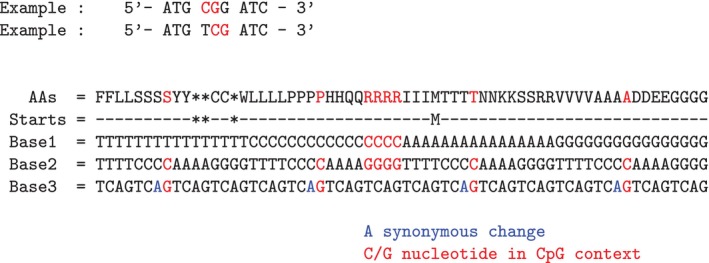
CpG sites within codons. Occurrences of CpG dinucleotides within codons of the standard genetic code. All 64 codons are shown (to be read from top, Base1, to bottom, Base3). CpG sites in codons at the first or second codon position are labeled in red. CpG dinucleotides, when methylated, can be subject to spontaneous deamination, resulting in a higher mutation rate in methylated CpG sites from CpG to TpG/CpA. 3rd codon positions of alternative synonymous codons potentially subject to spontaneous deamination are colored in blue. Stop (as asterisk) and Start codons (as M) are indicated in the row “Starts”. AAs, amino acids.

**FIGURE 3:**
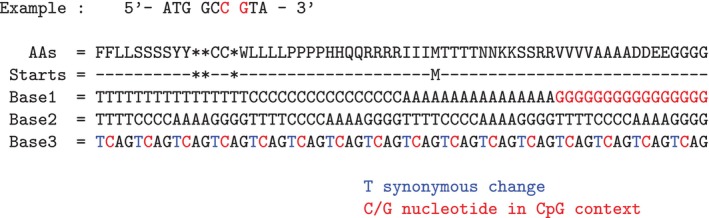
CpG sites across codons. All 64 codons are displayed, with Base1 at the top and Base3 at the bottom. CpG dinucleotides can form across codons when a codon ends with a C and the next codon starts with a G. Codon positions that may contribute to CpG sites at the first or third positions are marked in red. Methylated CpG dinucleotides are prone to spontaneous deamination, leading to an increased mutation rate (CpG → TpG/CpA). The third codon positions of synonymous codons, where such deamination may occur without altering the encoded amino acid, are highlighted in blue. Stop codons (indicated by an asterisk) and Start codons (indicated by M) are shown in the row labeled “Starts.” AAs, amino acids.

The evolutionary forces acting on protein coding DNA are more complex than on noncoding genomic DNA (Figure 1). CpG dinucleotides may occur in protein coding DNA, either within a single codon (Figure 2) or across codons (Figure 3), that is, at adjacent codons. They may also occur at exon‐intron boundaries, though this scenario is not discussed here.

#### 2.1.1 CpG Dinucleotides Within Codons

For the standard genetic code, CpG dinucleotides occuring at the first and second codon position (i.e., CpGpN, Figure 1B) are always coding for the amino acid arginine (R). DNA methylation driven mutations at first codon positions (CpG → TpG) in R encoding codons would lead to a change of the encoded amino acid (either to a C or W) or a stop codon. DNA methylation driven mutations at the second codon position (CpG → CpA) would also lead to changes in the encoded amino acids (H and Q changes). As these mutations result in nonsynonymous amino acid changes, there would be selection against DNA methylation driven mutations of CpG dinucleotides at codon position one and two. However, GC‐biased gene conversion would favor C or G containing variants, which would restore coding for arginine.

There are four amino acids that can be encoded by CpG dinucleotide containing codons at positions two and three: S, P, T and A (Figures 1C and 2). DNA methylation driven mutation at the third codon position (CpG → CpA) mutations would be affecting 4‐fold degenerate sites in all four cases, for example, these mutations would not involve a change of the encoded amino acid. GC‐biased gene conversion may nonetheless favor CpG sites over CpA sites (Table 1). On the other hand are (CpG → TpG) mutations at the second codon position would lead to a change in the amino acid, and may be removed by purfiying selection.

**TABLE 1:** Possible CpG SNP polymorphisms in protein coding DNA.

Mutation	Polymorphism	GC gene conversion acting	high mutation rate	Synonymous context at 3rd position	Synonymous context at 1st position
CpG					
1	↔ GpG	No	No	Possible	Never
2	↔ ApG	Yes	No	Possible	Possible
**3**	**↔ TpG**	**Yes**	**Yes**	**Always**	**Never**
**4**	**↔ CpA**	**Yes**	**Yes**	**Always**	**Never**
5	↔ CpT	Yes	No	Always	Never
6	↔ CpC	No	No	Always	Never

*Note:* Gene conversion tends to favor G and C nucleotides over T and A nucleotides at heterozygous sites, and this is therefore referred to as GC‐biased gene conversion (Gossmann et al. 2018; Bolvar et al. 2019). SNPs that may result from methylation‐driven mutations are indicated in bold.

#### 2.1.2 CpG Dinucleotides Across Codons

15 different amino acids have codons ending on a C base and could therefore potentially form CpG sites when following codons starting with a G. In all cases a CpG → TpG mutation would encode for the same amino acid, causing synonymous mutations and providing non‐CpG alternative codons for these dipeptides (Figures 1D and 3). There are also six amino acids that start with G codon, that could form the second site of the CpG dinucleotide. In all cases a methylation driven CpG to CpA mutation would lead to a change in the respective encoded amino acid, causing nonsynonymous mutations that may be exposed to purifying selection.

### 2.2 Any CpG Dinucleotide Can Be Replaced in the Standard Genetic Code

In summary, there are eight DNA codons that contain a within codon CpG site, and they encode for five amino acids (Figure 2). As noted above, four of these eight codons encode for arginine, which can, however, be encoded by two additional non‐CpG codons (AGG and AGA, Table 2). The other four amino acids that are encoded by a codon that contains CpG (Table 2) have at least three other non‐CpG containing codons. Even when forming dicodons, CpGs can always be avoided using a CpG free dicodon encoding for the same dipeptide (Figure 3). As a consequence, the standard genetic code allows in principle for any protein to be encoded without the use of CpG sites, although other restrictions on tRNA or base composition may still constrain this. For example, selection for high GC base composition may require codons to be composed of Gs and Cs only, which would require arginine codons to use CpG dinucleotides.

**TABLE 2:** Non‐CpG alternative encoding in the standard genetic code.

CpG site	Amino acid(s)	Alternative codon(s)	Single synonymous change	GC neutral alternative
**CG**N	R	AGA, AGG	partial	no
N**CG**	S, P, T, A	NCH	always	yes
NN**C G**NN	F, S, Y, C, L, P, H, R, I, T, N, V, A, D, G × V, A, D, E, G	NNT GNN	always	partially
None	M, Q, W	n.a.	n.a.	n.a.

*Note:* Codon compositions that would include a CpG dinucleotide (in bold), as well as alternative codons encoding the same amino acid/dipeptide. Three amino acids never occur within a CpG context. For DNA, *N* represents any of the four possible nucleotide bases adenine (A), thymine (T), cytosine (C), guanine (G). H stands for adenine (A), thymine (T), cytosine (C) consistent with the IUPAC nomenclature for DNA (Johnson 2010). GC neutral alternative indicates whether alternative codons would have the same number of G + C.

#### 2.2.1 A CpG Free Genetic Code

Under the assumption that there are 61 codons that code for an amino acid, one would need to exclude 8 codons that contain CpG sites (**CpG**pN and Np**CpG**) as well as 15 codons that potentially could form a CpG site with an adjacent codon (e.g., either GpNpN or NpNpC), this results in 38 available codons that can be used to form CpG free polypeptide chains. This leads to a substantial reduction of possible multi‐codons. For example, there are 3721 possible dipeptides (61 × 61) in the standard genetic code. When only the 38 CpG‐free codons are used only 1444 dicodons remain (a reduction of more than 60%).

#### 2.2.2 High CpG Content Polypeptide Chains

A polypeptide chain that contains a high amount of CpG sites, could be formed by poly‐R, poly‐S, poly‐T, poly‐P, and poly‐A chains. Methylation driven changes could replace CpG content with synonymous changes from all of these except the poly‐R. In contrast, particularly high levels of CpG could be formed with CpG sites that occur across a pair of dicodons, in particular [RxA]_*n*_ ([**CG**CG**CG**]_*n*_) and [AxN]_*n*_ ([G**CGCG**C]_*n*_).

### 2.3 CpG Features of Particular Amino Acids

There are only three amino acids that never occur within a CpG context, these are M and Q and W. All other amino acids may occur within a CpG context (Table 2). As noted previously, there is no single amino acid or dicodon that requires a CpG site. In most cases a single point mutation (Table 1) can lead to a synonymous change of a CpG codon to non‐CpG codon. At the third codon position a CpG → TpG/CpA is always synonymous. In contrast R is the only amino acid that may require 2 synonymous mutations to remove CpG sites: (**CG**T and **CG**C). There are, however, still two Arginine codons that can become CpG‐free with a single synonymous mutation–(**CG**A and **CG**G) and (CpG → ApG)–although neither of these are of types that would be driven by methylation (CpG → TpG/CpA). Hence R encoding codons should be particularly unlikely to be replaced by CpG‐free alternatives.

## 3 Material and Methods

### 3.1 Data Download

Protein coding DNA sequences were downloaded in fasta format from NCBI refseq (https://ftp.ncbi.nlm.nih.gov/genomes/refseq/) for one ray‐finned fish, *Gasterosteus aculeatus* , the threespine stickleback (Jones et al. 2012), one frog, *Xenopus tropicalis* (Hellsten et al. 2010) as well as two birds ( *Parus major* , great tit (Laine et al. 2016) and *Gallus gallus* , chicken (International Chicken Genome Sequencing Consortium 2004)) and two mammalian species, *Homo sapiens* , human (Lander et al. 2001) and *Mus musculus* , mouse (Mouse Genome Sequencing Consortium 2002) (Table 3). We did not opt for more fish genomes because the frequency of CpG dinucleotides is seemingly higher in fish than in other vertebrates (Jabbari and Bernardi 2004). To reduce the number of splicing variants, only genes with assigned gene symbols were considered for the analysis. The genomic data was downloaded from NCBI refseq genomes.

**TABLE 3:** Summary of the genome assemblies used for this analysis.

Species	Common/lay name	Assembly
*Homo sapiens*	Human	GCF_000001405.39_GRCh38.p13
*Mus musculus*	Mouse	GCF_000001635.27_GRCm39
*Parus major*	Great tit	GCF_001522545.3_Parus_major1.1
*Gallus gallus*	Chicken	GCF_016699485.2_bGalGal1.mat.broiler.GRCg7b
*Gasterosteus aculeatus*	Stickleback	GCF_016920845.1_GAculeatus_UGA_version5
*Xenopus laevis*	Frog	GCF_017654675.1_Xenopus_laevis_v10.1

*Note:* Genome and cds sequence files were obtained from NCBI refseq.

### 3.2 CpG Content

As CpG content may vary depending on base composition and strand bias (Box 1) these factors need to be taken into account when measuring CpG content. For example for CpG islands, regions in the genome with an excess of CpG sites, are defined as regions with an observed/expected ratio of CpG to GpC larger than 0.6 (Gardiner‐Garden and Frommer 1987). However, the CpG/GpC ratio may become very skewed if the stretch of DNA under consideration is small. Therefore, we slightly adopt this measure and quantify CpG content as the proportion of CpG sites of palindromic C/G containing dinucleotides in a stretch of DNA (for genomic fragments of 5 kb size, or smaller if a scaffold was smaller than 5 kb):

(1) $$\mathrm{CpG}\;\text{content}=\frac{\#\mathrm{CpG}}{\#\mathrm{CpG}+\#\mathrm{GpC}}$$

hence

(2) 0≤CpGcontent≤1

### 3.3 Protein Coding DNA and Genomic DNA

We only considered genes with assigned gene symbols or gene names for the analysis (McCarthy et al. 2023; Seal et al. 2022) (e.g., excluded genes with species‐specific genomic location index) to avoid including partial genes and pseudogenes in the analysis. This also reduced the number of splicing variants included and controlled for potential annotation quality differences between the different genomes (Yusuf et al. 2020). For technical reasons, we also excluded genes or gene fragments that lacked both CpG and GpC dinucleotides, as this would result in a zero denominator. For the gene fragment analysis, we also excluded genes that were shorter than 200 nucleotides to ensure that the start and end of the coding sequences were distinct. Because the genome assemblies for each species were of varying quality, with some species having fragmented chromosome assemblies, we calculated CpG content for genomic fragments of 5 kb size (or smaller if a scaffold was smaller than 5 kb) for each species. Statistics were then obtained on a gene‐by‐gene or fragment‐by‐fragment basis.

### 3.4 Statistical Analysis

**FIGURE 4:**
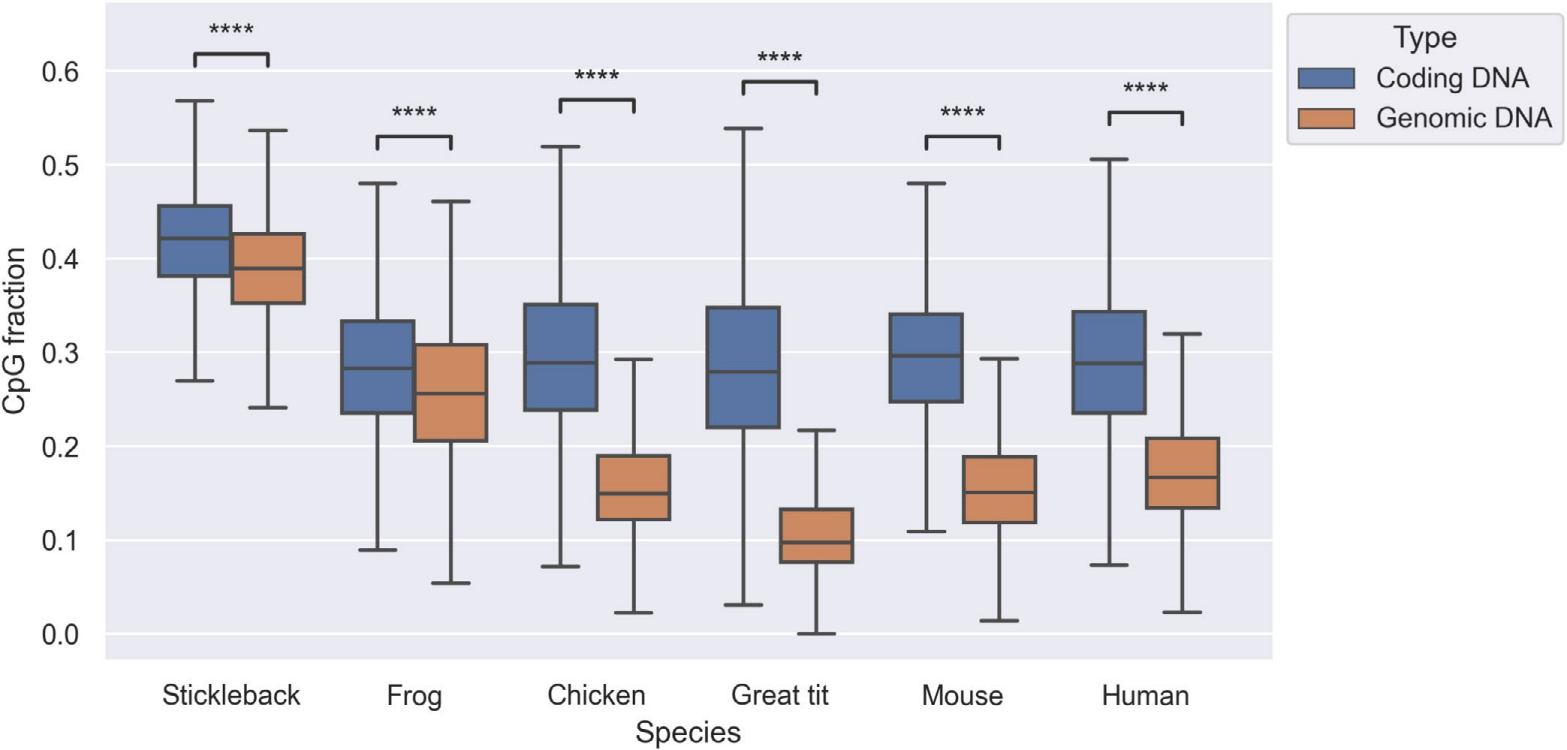
CpG content in different vertebrate species summed across the genome. CpG content is measured as the fraction of CpG sites in GpC and CpG dinucleotides. Shown are protein coding DNA (Coding DNA, blue) and the entire genomes (Genomic DNA, orange). Statistical differences were assessed with a Mann–Whitney‐*U* test, ****p ≤ 10^−4^.

**FIGURE 5:**
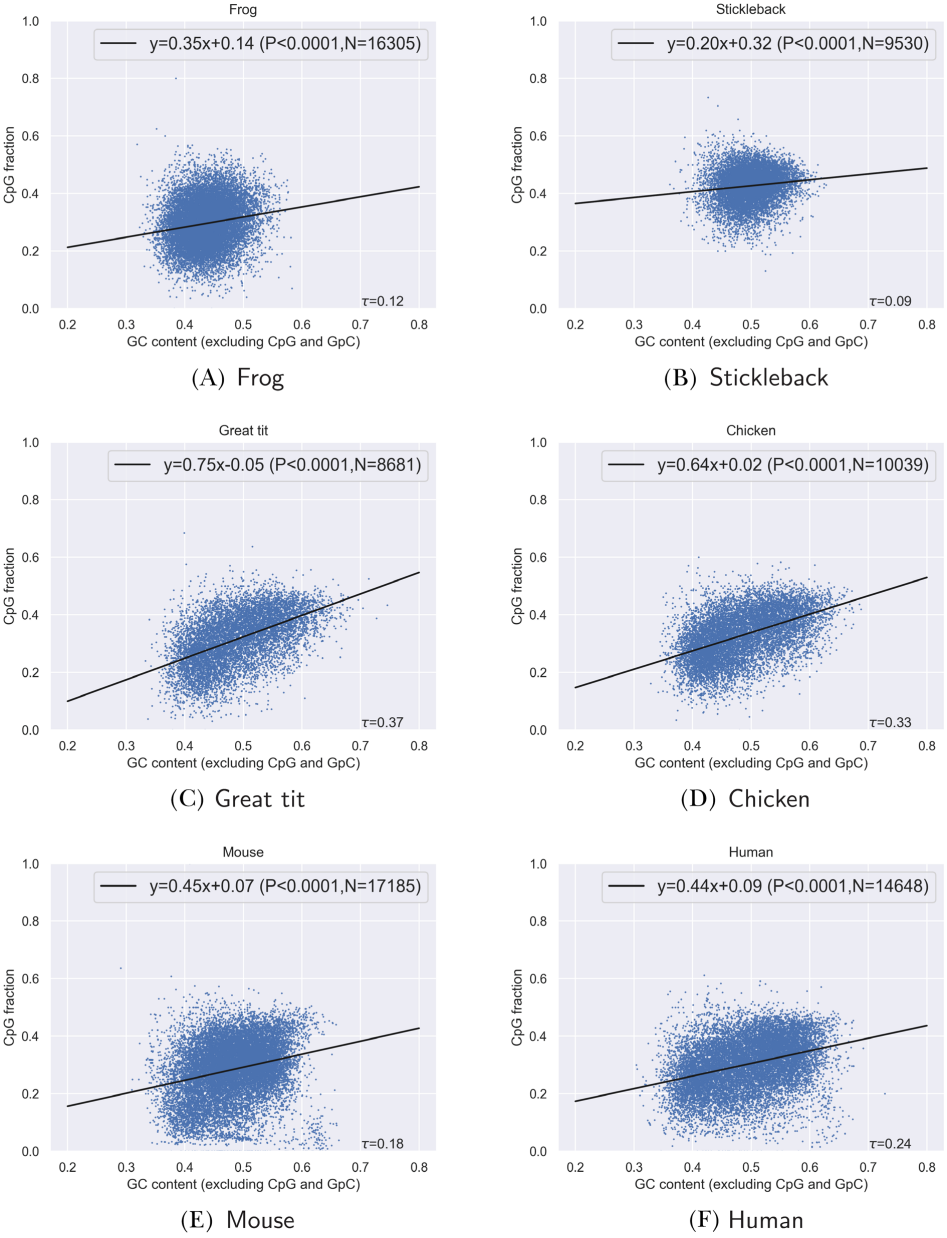
Correlation of CpG content with GC content in protein‐coding genes for six species (A–F). For all cases there is a positive correlation between CpG content and GC content. Note that genes smaller than 150 nucleotides and larger 2000 nucleotides were excluded from the analysis. The correlation coefficient *τ* is given as well as the parameters of the linear regression line and its associated *p* value. *N* denotes the number of genes in the analysis.

**FIGURE 6:**
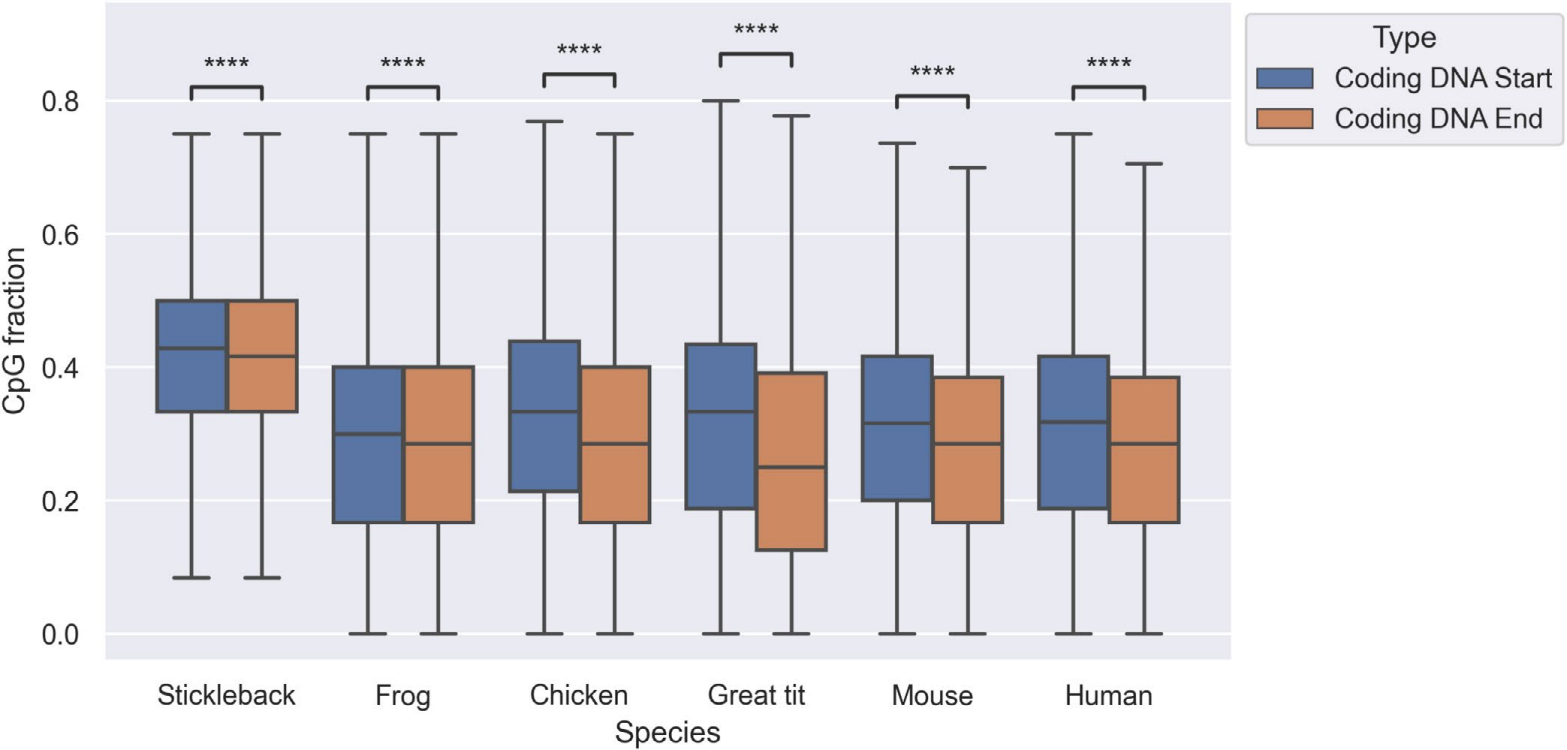
Coding CpG contents at the start and end of the coding DNA in different vertebrate species. CpG content is measured as the fraction of CpG sites in GpC and CpG dinucleotides. The first 99 coding basepairs (N‐terminal end) and the last 99 coding basepairs (C‐terminal end) of each gene were used. Statistical differences were assessed with a paired Wilcoxon rank test, ****p ≤ 10^−4^.

Statistical analyses were performed with the SciPy (Virtanen et al. 2020) and NumPy (Harris et al. 2020) packages in Python or using the statistical tests implemented in Webgestalt (Liao et al. 2019). Statistics were retrieved for each gene for coding DNA and each 5 kb fragment for genomic DNA. To compare statistical significance we applied a Mann–Whitney‐*U* test (Figure 4), a linear regression and Kendall *τ* (Figure 5) and a Wilcoxon paired rank test (Figure 6). Statistical annotations of Figures 4 and 6 were conducted with the package *statannotations* (Charlier et al. 2022) using the default legend for *p* values.

### 3.5 Data Visualization

All data were analyzed with Python 3.8 with respective NumPy, Scipy, matplotlib packages. Data visualization was done with matplotlib and seaborn (unless otherwise stated) and respective packages. The only exception to this are the upset plots, which were made in R 4.3.3 using the ggVennDiagram package and the permutation tests, which were conducted in R with the package “BiocManager”.

### 3.6 Gene Ontology Analysis

We used the 2019 version of WebGestalt (https://2019.webgestalt.org/) (Liao et al. 2019) to perform an Over‐Representation Analysis (ORA) with the functional database set to “geneontology” and the “biological process non‐redundant data,” based on *Homo sapiens* annotation. For this analysis, we combined the top 100 CpG poorest and richest genes across six vertebrate species. The gene ID type was specified as “gene symbol,” and the human genome (“genome”) served as the reference gene list. The results were qualitatively similar whether we analyzed single species or used species‐specific gene lists as references (see Figure S1 for coding DNA with high CpG content). We submitted our jobs with the following advanced parameter settings: the minimum number of genes required for a category was set to 10, and the significance level was defined using a false discovery rate (FDR) of 0.05. All other parameters remained unchanged (maximum number of genes 2000; “BH” for multiple test adjustment; expected number of categories from set cover set to 10; number of categories visualized set to 40; and continuous color used in the DAG). Additionally, we conducted a pathway‐level analysis (KEGG) using the same parameters.

### 3.7 Chromatin State Analysis

For the chromatin state analysis we downloaded data from the ENCODE Project Portal website. ENCODE provides chromatin state segmentations for various human cell types, and we filtered datasets available in GRCh38 for “ChromHMM”. Specifically, we focused on data entry ENCFF343KUN. To obtain the genomic locations of the 100 human CpG poorest and CpG richest genes, we uploaded the respective gene lists into the UCSC Table Browser (https://genome.ucsc.edu/cgi‐bin/hgTables) and set the assembly to hg38 (GRCh38). We then selected the gene track (i.e., Gencode V47) and applied filters to include our gene list. Finally, we used a customized R script to load two BED files (i.e., gene coordinates and chromatin states), find their overlapping regions, and perform a permutation‐based significance test using the regioneR package to assess whether the observed overlap is greater than expected by chance.

## 4 Results

The complex interplay between the genetic code, methylation‐driven mutation, and GC‐biased gene conversion raises questions as to the abundance of CpG sites in coding sequences. Due to their regulatory potential and hypermutability, CpG sites may be either deprived or enriched in coding domain sequences. For this reason, we tested features of CpG abundance in protein‐coding DNA in six vertebrate species, including a fish, a frog, two bird species, and two mammalian species (Table 3). We explicitly evaluate CpG content in coding domain sequences using genome‐wide patterns of CpG abundance as a control. We chose at least one representative genome of the three classes Mammalia, Aves, and Reptilia, as well as one ray‐finned fish genome.

### 4.1 CpG Content in Protein Coding DNA is Higher Than the Overall Genome

#### 4.1.1 CpG Content Heterogeneity Across Protein Coding DNA

As CpG frequency in a given genomic region may depend on the GC content (Box 1) which varies across the genome, as well as the length of the region, we used a normalized measure of CpG content that adjusts for these potential sources of bias: the ratio of CpG to GpC dinucleotides (see Equation (1) in ‘Materials and Methods’). The frequency of CpG dinucleotides varies between protein coding genes and the overall genome for vertebrates (Figure 4). For all six species, it is lower in overall genomic DNA relative to protein coding DNA (this was highly significant for all six pairwise comparisons, *p* < 10^−4^, MWU‐test). The magnitude of this difference is heavily influenced by species: for the mammalian and avian species, the difference is around twice as much, while for frog and stickleback the difference between protein coding DNA and genomic DNA is more modest.

#### 4.1.2 CpG Content and Base Composition

CpG content may be driven by overall GC content (Box 1). To understand whether the CpG composition is correlated with base composition we obtained CpG content and GC content (excluding CpG and GpC sites) of protein coding sequences and found a positive correlation in all species, albeit with variable slopes (Figure 5, Kendall's *τ* between 0.09 for stickleback and 0.37 for great tit). We also note that genes with very high CpG content in coding domains tend to occur in regions with intermediate GC content, which would suggest that these are not necessarily a by‐product of high GC nucleotide composition.

### 4.2 CpG Content in Protein Coding DNA is Higher Near TSS

#### 4.2.1 CpG Content Heterogeneity Within Genes

The frequency of CpG dinucleotides varies between coding domains but there might also be variation within these. To test this we obtained the CpG content at the first 99 sites at the start (5′ end, N‐terminal) and 99 sites at the end (3′ end, C‐terminal) of protein coding sequences within genes (Figure 6). Indeed, for all six species there is a significant difference between CpG content at the start and the end of coding DNA (highly significant for all six pairwise comparisons, *p* < 10^−4^, Wilcoxon paired rank test). CpG content is higher at the start relative to the end, and this pattern is strongest in the mammalian and avian species.

### 4.3 Most Frequent and Most Rare Dicodons Contain CpG Sites

#### 4.3.1 CpG Sites in Dicodons

As shown above, in the standard genetic code any CpG containing dicodon may be avoided when coding for any polypeptide chain. We therefore examined whether particular dicodons would occur less frequently than expected based on their codon abundances. As a safeguard we also look at all overrepresented dicodons, as we might expect enrichment in TpG sites as a consequence of the methylation‐driven mutations. With the exception of the stickleback, the three least abundant dicodons are those containing across codon CpG sites (Table 4). Interestingly, the most abundant dicodons also contain many CpG sites—often at the second and third position, and encode for di‐alanin and di‐prolin in particular.

**TABLE 4:** Top 3 under‐ and overrepresented dicodons in 6 vertebrate species in protein coding DNA.

Species	Enrichment	Dicodon	AA1	AA2	Enrichment	Dicodon	AA1	AA2
Human	0.110	GT**CG**AA	V	E	6.146	G**CG**G**CG**	A	A
Human	0.113	CT**CG**AA	L	E	5.909	C**CG**C**CG**	P	P
Human	0.141	GG**CG**AA	G	E	3.415	TGCTGC	C	C
Frog	0.106	**CGCG**AA	R	E	4.453	G**CG**G**CG**	A	A
Frog	0.115	CT**CG**AA	L	E	2.761	ATGG**CG**	M	A
Frog	0.143	GT**CG**AA	V	E	2.723	AGCAGC	S	S
Mouse	0.109	GT**CG**AA	V	E	6.591	G**CG**G**CG**	A	A
Mouse	0.110	CT**CG**AA	L	E	5.410	C**CG**C**CG**	P	P
Mouse	0.148	GT**CG**AG	V	E	3.568	TGCTGC	C	C
Great tit	0.059	**CGCG**AA	R	E	8.959	G**CG**G**CG**	A	A
Great tit	0.094	CT**CG**AA	L	E	6.445	C**CG**C**CG**	P	P
Great tit	0.102	GT**CG**AA	V	E	3.683	C**CG**G**CG**	P	A
Chicken	0.103	**CGCG**AA	R	E	7.396	G**CG**G**CG**	A	A
Chicken	0.106	GT**CG**AA	V	E	5.876	C**CG**C**CG**	P	P
Chicken	0.127	TT**CG**CA	F	A	3.712	**CG**G**CG**G	R	R
Stickleback	0.089	GCTAGG	A	R	3.950	CCTCCT	P	P
Stickleback	0.095	CCTAGG	P	R	3.594	CCTCCA	P	P
Stickleback	0.101	CCTAGC	P	S	3.393	G**CG**G**CG**	A	A

*Note:* CpG sites are highlighted in bold. Many of the dicodons contain CpG sites. AA1 and AA2—first and second amino acids, respectively, of the dipeptide that results from the translation of the dicodon.

### 4.4 Functional Implications of CpG Sites in Protein Coding DNA

**FIGURE 7:**
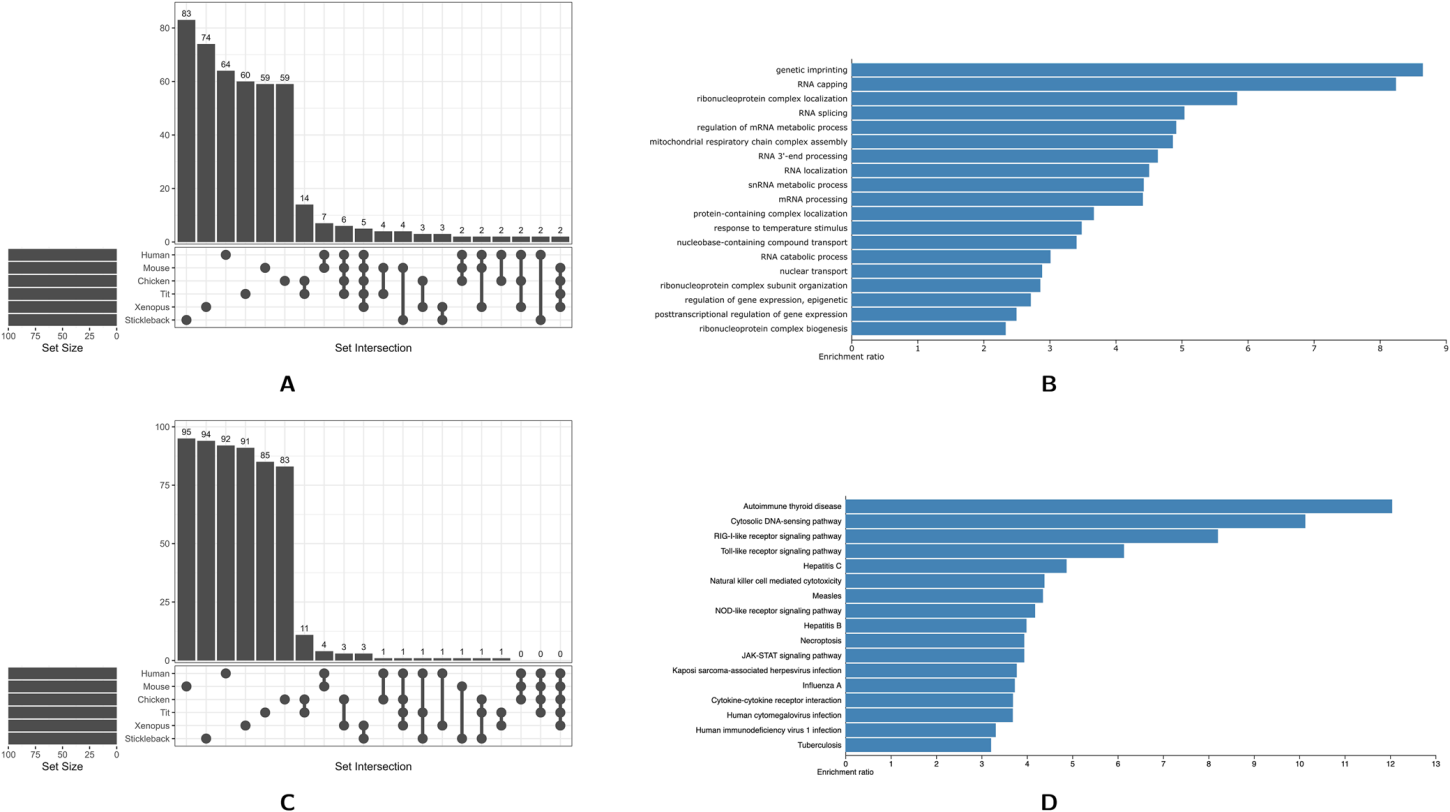
Functional association of genes with high and low CpG content in six vertebrate species. The 100 most and least CpG rich genes across six vertebrate species were combined and analysed for gene ontology overrepresentation. (A) Upset plot of unique and shared genes of the 100 highest CpG dinucleotides in each species. (B) Enrichment categories for the high CpG rich genes with FDR < 0.05 visualised through the WebGestalt server. (C) Upset plot of unique and shared genes of the 100 lowest CpG dinucleotides in each species. (D) KEGG Pathway Enrichment categories with FDR values < 0.05 for the low CpG rich genes visualised through the WebGestalt server.

To obtain potential insights into the functional implications of CpG dinucleotide abundance in protein coding genes, we conducted functional enrichment analysis using Gene Ontology (GO) terms. For this, we identified the top 100 genes with the highest and lowest CpG to GpC ratio and combined them across all six vertebrate species. This yielded 473 genes for CpG rich genes and 568 genes for CpG poor genes. We then conducted a functional overrepresentation analysis using the human genome as a reference set, although species‐specific analyses with species‐specific reference sets gave similar results (Figure S1), GO enrichment for the functional categories genetic imprinting and response to temperature stimuli were pan‐species. It is also noteworthy that most of the identified genes with high/low CpG composition were species specific (Figure 7A,C).

#### 4.4.1 Functional Chromatin Associations of CpG Rich and CpG Poor Genes in Humans

**FIGURE 8:**
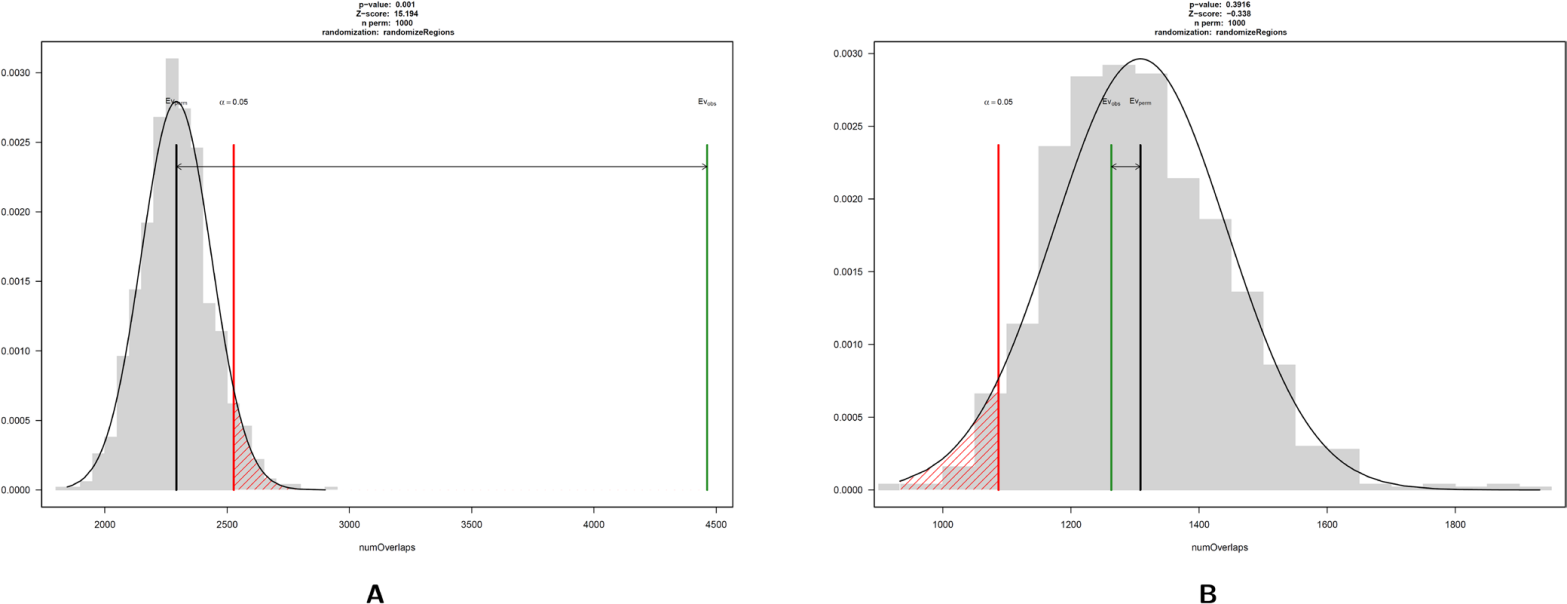
Permutation test of genomic regions overlap between gene bodies and chromatin state in humans. Chromatin state was obtained from kidney epithelial cells deposited in the ENCODE database (ENCFF343KUN). Other tissues were very similar (results not shown). (A) The 100 most CpG‐rich genes show a significant enrichment in regions with functional chromatin states. (B) The 100 CpG‐poorest genes show no significant overlap with functional chromatin state.

We investigated the overlap of genes (i.e., gene bodies) and genomic regions with a functional chromatin annotation in humans. Specifically, we harvested data from the ENCODE project on kidney epithelial cells, although other tissue types showed a similar pattern. While CpG rich genes were significantly enriched in regions of functional chromatin states (*p* < 0.001, Figure 8A), there was no such overlap observed for the CpG poorest genes (*p* = 0.39, Figure 8B).

#### 4.4.2 Functional Association of Most CpG Rich Genes and CpG Poor Genes

We found nineteen gene categories that are overrepresented in our dataset of CpG rich genes at FDR < 0.05. Most of these categories can be related to gene regulation, including epigenetic processes such as genetic imprinting, response to temperature stimuli (Table 5) and regulation of RNA/mRNA (Figure 7B). The gene categories for genetic imprinting and RNA capping, which include several RNA polymerase II subunits, exhibit an enrichment of more than 8‐fold. On the pathway level, “Spliceosome” (hsa03040) and Huntington disease (hsa05016) had an FDR < 0.01 for the CpG richest genes.

**TABLE 5:** List of genes for two enriched GO terms among CpG rich genes.

Gene symbol	Gene name	Entrez gene	Functional category
DPPA3	developmental pluripotency associated 3	359,787	Genetic imprinting
GNAS	GNAS complex locus	2778	Genetic imprinting
KCNQ1	potassium voltage‐gated channel subfamily Q member 1	3784	Genetic imprinting
MECP2	methyl‐CpG binding protein 2	4204	Genetic imprinting
PCGF6	polycomb group ring finger 6	84,108	Genetic imprinting
ADM	adrenomedullin	133	Temperature stimulus
CASQ1	calsequestrin 1	844	Temperature stimulus
CETN1	centrin 1	1068	Temperature stimulus
CIRBP	cold inducible RNA binding protein	1153	Temperature stimulus
COX2	cytochrome c oxidase subunit II	4513	Temperature stimulus
CRYAB	crystallin alpha B	1410	Temperature stimulus
DNAJB1	DnaJ heat shock protein family (Hsp40) member B1	3337	Temperature stimulus
EIF2B1	eukaryotic translation initiation factor 2B subunit alpha	1967	Temperature stimulus
HSBP1L1	heat shock factor binding protein 1 like 1	440,498	Temperature stimulus
HSPB8	heat shock protein family B (small) member 8	26,353	Temperature stimulus
HTR1B	5‐hydroxytryptamine receptor 1B	3351	Temperature stimulus
PDCD6	programmed cell death 6	10,016	Temperature stimulus
RPA3	replication protein A3	6119	Temperature stimulus
SUMO1	small ubiquitin‐like modifier 1	7341	Temperature stimulus
TCIM	transcriptional and immune response regulator	56,892	Temperature stimulus

*Note:* Five genes from the genetic imprinting category (GO:0071514; total size = 27, overlap = 5, expect = 0.58, enrichmentRatio = 8.65, *p*‐value = 2.387e−4, FDR = 1.193e−2) and 15 genes from the response to temperature stimulus category (GO:0009266; size = 201, overlap = 15, expect = 4.30, enrichmentRatio = 3.49, *p*Value = 2.862e−5, FDR = 2.027e−3).

We identified five categories for CpG‐poor genes that are enriched in our dataset. Processes related to skin and epidermis development (GO:0043588 and GO:0008544) were highly enriched, suggesting a role in epithelial differentiation. Also immune‐related functions—such as natural killer cell activation (GO:0030101) and the response to double‐stranded RNA (GO:0043331)—were significantly enriched. Additionally, enrichment for peptide cross‐linking (GO:0018149) points toward alterations in protein interaction dynamics. Sixteen KEGG pathways were significantly enriched in the dataset, with a clear emphasis on immune and antiviral responses. Pathways include autoimmune thyroid disease, cytosolic DNA‐sensing, RIG‐I‐like receptor signaling, as well as several viral infection pathways (e.g., Hepatitis C, Influenza A, HIV‐1), underscoring an involvement of innate immune processes (Figure 7D).

## 5 Discussion

Here we have shown that CpG motifs potentially play a special role in protein coding DNA. Generally speaking, high mutation rates at methylated CpG sites may lead to loss of these sites in genomic, including protein coding, DNA (Figure 1). Conversely, GC‐biased gene conversion could maintain these sites and allow them to be favored over (A/T)pG or Cp(A/T) sites, if it allows for reversal of a non‐synonymous amino acid change in the context of purifying selection. However, the interplay of epigenetics, mutation, biased conversion, and selection is highly context dependent in protein coding DNA (Figure 1B–D). In our theoretical analyses, we show that the standard genetic code is composed in such a way that any protein chain could be encoded without the use of CpG sites. Indeed, CpG dinucleotides are the only facultative dinucleotides in the eukaryotic genetic code, making them uniquely capable of avoiding the evolutionary constraints typically imposed on coding DNA. However, limitations on codon availability and base composition may still expose these dinucleotides to selection, as non‐CpG free exchange is not always base neutral. Importantly, it has also been observed that the codon composition in vertebrates is realized in such a way that nonsense mutations tend to be avoided (Kanaya et al. 2001; Schmid and Flegel 2011), and other code‐based constraints may still apply.

To understand the consequences of the complex interplay of nucleotide composition and evolutionary forces acting on protein coding DNA, we investigated the genomes of six vertebrate species. We first asked whether CpG sites are more common in protein coding DNA compared to the rest of the genome and whether there is heterogeneity in CpG content within genes. Indeed, we find that CpG sites in protein coding DNA are enriched and more common than expected relative to the genome at large, and we also confirm substantial variation in CpG dinucleotide abundance across and within genes (Bricout et al. 2023). In particular, the 5‐prime end of coding domain sequences is enriched in CpG sites, potentially due to low methylation near transcription start sites or because exon 1 methylation often has strong effects on transcriptional control (Derks et al. 2016; Hodgkinson and Eyre‐Walker 2011; Fang et al. 2023).

We then examined what the over‐ and underrepresented dicodons in protein coding DNA are and whether they contain CpG dinucleotides. We show that CpG sites tend to be enriched in certain proteins. Dicodons encoding for di‐proline and di‐alanine are particularly enriched, which is likely due to the fact that they are part of special amino acid repeats (Barik 2017) that use simple codon repeats and avoid the formation of certain RNA secondary structures. Gene body methylation is generally high in vertebrates (Derks et al. 2016), except for 5‐prime regions. For avian neuronal tissue, highly expressed genes tend to show lower levels of methylation, as do CpG islands (Laine et al. 2016). All these could be contributing factors as to why some proteins show an enrichment in CpG sites: Lower methylation levels would result in lower rates of mutation while purifying selection and optimization for optimal codons (Hershberg and Petrov 2009) maintain high CpG abundance (Figure 1). Enrichment of CpG content may also favor open chromatin and transcription (Angeloni and Bogdanovic 2021) and help to avoid adenine‐rich alternative codons, which may compromise translation (Ruggiero and Boissinot 2020; Perepelitsa‐Belancio and Deininger 2003). Interestingly, coding genes with high levels of CpG sites tend to show modest levels of GC content, which supports these notions. Exploration of methylation patterns in germline tissues will likely be important for disentangling the relative roles of methylation‐driven mutations, base composition, and chromatin state (Figure 8) on the evolution of CpG content in coding regions (Messerschmidt et al. 2014).

Finally, we asked whether there is any functional enrichment of genes that show extreme CpG content in their protein coding DNA. We find strong functional enrichment for CpG‐rich genes related to gene expression and regulation. This is in line with previous evidence that in species with highly methylated genomes, strong pro‐epigenetic selection (i.e., selection favoring the capacity to be methylated) acts on some CpG‐containing genes, particularly DNA‐binding transcription factors involved in developmental regulation (Branciamore et al. 2010). If, indeed, higher CpG content of protein coding DNA is maintained by pro‐epigenetic selection, this could provide clues as to the hitherto elusive functions of gene body methylation. However, in our setup, it is difficult to tease apart selection on CpG sites from other potential sources, such as selection on protein‐changing substitutions, selection on optimal codon usage, avoidance of deleterious changes to RNA 3D structure, or other intertwined functional implications (Hu et al. 2023; Ord et al. 2023). Taking into account the action of selection (Figure 1), it is perhaps not surprising that CpG frequency is higher in protein coding DNA compared to the rest of the nuclear genome. Due to the features of the genetic code, there is a complex interplay of mutation bias, selection pressure, codon optimization, and functional constraints in maintaining CpG‐rich regions within protein coding genes. Notably, genes exhibiting high CpG content are functionally linked to regulation, RNA expression, and potential epigenetic regulation across vertebrate species, underlining the intricate relationship between natural selection, mutational bias, and epigenetics in shaping gene properties.

Our findings have evolutionary applications in conservation, breeding, and genome engineering. The enrichment of CpG sites in genes involved in gene regulation and stress responses suggests that CpG content could be a valuable marker for assessing adaptive potential in natural populations. Conservation programs might be able to leverage this information to prioritize populations with higher evolutionary resilience. Additionally, CpG content dynamics can guide breeding strategies by identifying genes linked to traits such as stress tolerance and growth optimization. The identification of CpG‐free codons and CpG‐rich loci provides practical insights for synthetic biology and genome engineering, such as when targeting specific genes with CpG clusters by programmable DNA binding proteins (Clark et al. 2016; Buchmuller et al. 2021; Jung et al. 2023). These findings highlight the complex interplay of epigenetic regulation, mutation bias, and selection in shaping the evolution of vertebrate genomes. Future research on CpG methylation patterns in germline and somatic tissues, across a broader range of species, and within specific contexts such as gene expression and phenotypic plasticity, will be crucial for deepening our understanding of how these mechanisms shape genome evolution and drive adaptation across diverse ecological and selective landscapes. Expanding on this, the potential for more detailed analyses across species and even populations offers exciting opportunities to explore adaptive plasticity and to investigate whether gene body methylation plays a role in its evolution, further enriching our understanding of these complex processes.

## Conflicts of Interest

The authors declare no conflicts of interest.

## Supporting information


Figure S1

## Data Availability

All data used in this work is publicly available through NCBI and associated data repositories.
