# Supplementary material for: The CpG Landscape of Protein Coding DNA in Vertebrates
Source: Evol Appl. 2025 May 4;18(5):e70101. doi: 10.1111/eva.70101 (PMC12050414; doi:10.1111/eva.70101)
Supplement: Supplementary file 1 — Figure S1 [file EVA-18-e70101-s001.pdf]

Supplementary Material

Supplementary Files

https://github.com/tgossmann/CodingCpG/raw/refs/heads/main/Suppl_data.zip

- Gene identifiers for 100 top CpG rich genes for 6 species (high100.zip)
- Gene identifiers for 100 top CpG poor genes for 6 species (low100.zip)
- Permutation test for bed file overlap (compare2bed.R)

Supplementary Figures

- Figure S1

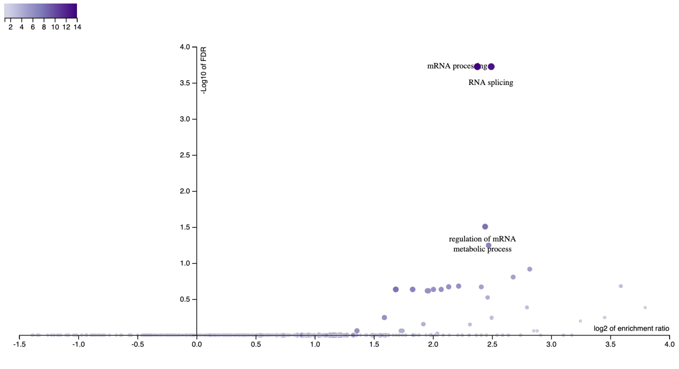

(a) Frog

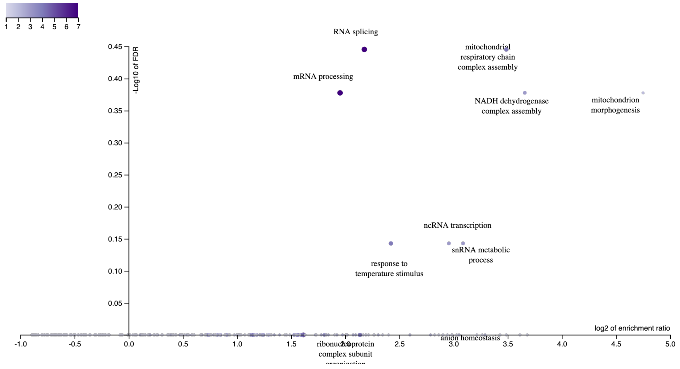

(b) Stickleback

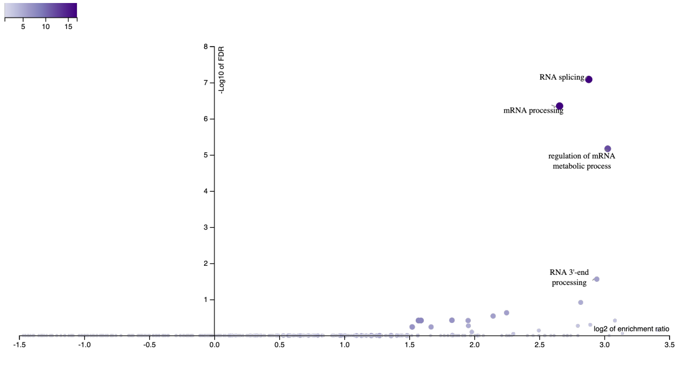

(c) Great tit

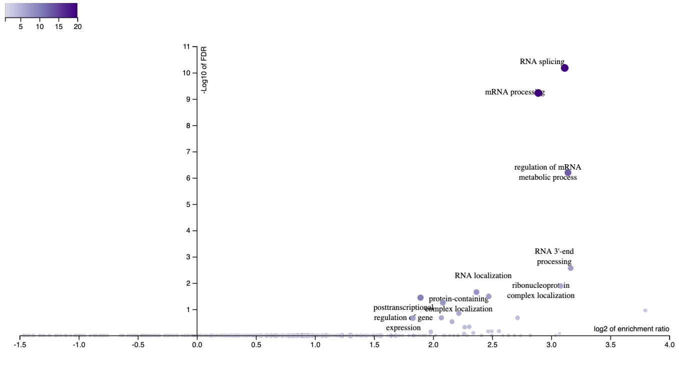

(d) Chicken

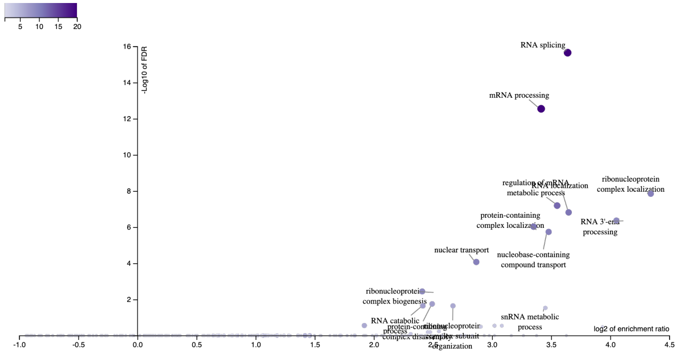

(e) Mouse

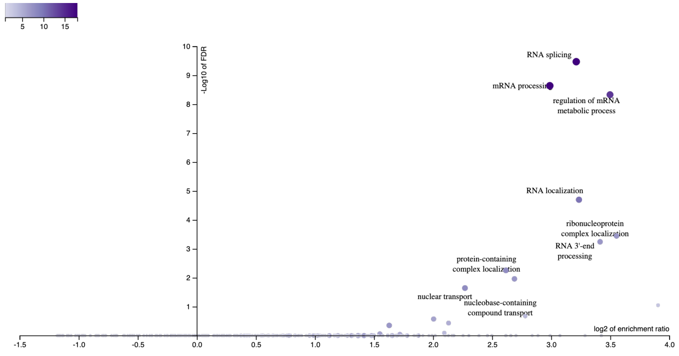

(f) Human

Figure S1: **Enrichment Results illustrated as volacano plot for protein coding genes with high CpG content for six species (a)-(f).**
